# The role of traditional birth attendants and problem of integration with health facilities in remote rural community of West Omo Zone 2021: exploratory qualitative study

**DOI:** 10.1186/s12884-022-04753-5

**Published:** 2022-05-20

**Authors:** Aychew Kassie, Alemnew Wale, Desalegn Girma, Hailemariam Amsalu, Mastewal yechale

**Affiliations:** 1 Deparetment of Nursing, College of Medicine and Health Science, South Nation, Nationality and People Region, Mizan Tepi University, Mizan, Ethiopia; 2 Deparetment of Midwifery, College of Medicine and Health Science, South Nation, Nationality and People Region, Mizan Tepi University, Mizan, Ethiopia; 3 Deparetment of Biomedical, College of Medicine and Health Science, South Nation, Nationality and People Region, Mizan Tepi University, Mizan, Ethiopia

**Keywords:** Traditional birth attendant, Role, Integration, Rural community, West Omo, Ethiopia

## Abstract

**Background:**

Mothers in rural Ethiopian communities prefer giving birth at home. In developing countries, traditional birth attendants play an important role in reducing the maternal mortality rate. In Ethiopia, however, their role during pregnancy, childbirth, the postnatal period, and their integration with health professionals is not clearly defined. This study aimed to explore the role of traditional birth attendants in feto-maternal care during pregnancy, childbirth, and the postnatal period, and integration with health professionals in the West Omo Zone, southern Ethiopia.

**Methods:**

A qualitative descriptive design was used with triangulation of methods and data sources. We conducted in-depth interviews with traditional birth attendants, key informant interviews with health care professionals and community or religious leaders, and two focus group discussions with multiparous pregnant women. Each interview and focus group discussion was tape-recorded and the data obtained were transcribed and translated into English for analysis. The analysis was done based on thematic analysis framework.

**Results:**

Traditional birth attendants stated that they used herbal remedies to treat nausea and vomiting, decrease pain during labor, and increase pregnant women's desire to push. The absence of incentives for their work, shortage of logistics, and lack of training was mentioned as challenges to the continuity of their roles. All study participants explained the importance of training traditional birth attendants on maternal and child health in rural communities. However, health care professionals reported that few traditional birth attendants advised mothers about traditional practices such as milk tooth extraction and uvulectomy.

**Conclusion and recommendation:**

Traditional birth attendants continued their roles despite the existing challenges. There was no integration between TBA and the formal health care system. The need for training traditional birth attendants has been emphasized by all study participants and its impact on reducing feto-maternal death was recognized by health care professionals. Therefore, the federal ministry of health should works better for the development of TBAs to scale up their skills across all regions in the country.

## Introduction

According to the World Health Organization (WHO) report of 2015, about 99% of maternal deaths occurred in developing countries, and half of these occurred in sub-Saharan Africa. The WHO indicates that a successful strategy to reduce the global maternal mortality rate has to increase the number of trained and educated individuals to help and care for the mother during pregnancy, delivery, and the postnatal period [1, 2].

The integration of traditional birth attendants (TBAs) within the country’s health care system, particularly in the field of maternal and child healthcare is very helpful in developing countries [3].

One advantage of traditional birth attendant utilization is that they are already connected to the communities they serve. While these women are untrained and uneducated in the standards set by the formal health care system, they have already established a trusting relationship with women in need of a birth attendant [4]. Moreover, logistical problems associated with skilled birth attendants such as lack of travel or transportation, expensive transportation services, and difficult geographic areas are overcome by traditional birth attendats [5].

In rural parts of Africa, 60% to 90% of pregnant women use TBAs during childbirth [6]. If these TBAs could be trained, the formal health care system would not need to exert resources in these areas [7]. Moreover, their close relationship with the community they live in plays an important role in bridging communities and health care systems [8, 9].

Evidence has shown the effectiveness of interventions such as training and support of TBAs in improving maternal and newborn health outcomes while reducing perinatal, neonatal, and maternal mortality [10–13]. Other studies have also shown that the integration of TBAs with the health care system has increased the skilled birth attendance rate [14, 15].

However, negative attitude towards TBAs, financial issue for TBAs, long distance to health facilities, transportation problems, and delay in seeking care by women were some of the barriers for integration with the health care system [16, 17].

Studies have shown that people often prefer TBA to a trained midwife, especially when the midwife is a young, unmarried girl without children [18]. TBAs not only provide technical assistance but also attend to and support the mother during the entire childbirth process and thereafter. The work of TBAs is adapted and strictly bound by the social and cultural matrix, and their practice and belives are linked to the community they live [19–21].

Further studies are needed to investigate the role of TBAs in maternal health care and their integration with health facilities in remote rural communities. This study aimed to explore the role of TBAs during pregnancy, childbirth, and the postnatal period and their integration with health care professionals in the remote rural communities of West Omo Zone in southern Ethiopia.

## Methods

### Study setting

This study was conducted in the West Omo Zone of Southern Ethiopia. It has eight districts and 116 kebeles (112 rural and 4 urban), with a total population of 272,943 people and 137,918 (50.53%) rural residents. The total number of households was 55,703, of which more than 60% lived in pastoral communities. This zone has one primary hospital 11 health centers and 96 health posts.

### Study design

An exploratory qualitative study approach was used. Data were collected through individual interviews with TBAs, community or religious leaders, and HCPs working at governmental health facilities in the study area. FGDs have also been conducted with multiparous pregnant women. Triangulation is recommended for qualitative studies to ensure an inclusive understanding of the studied phenomena [22]; therefore, we used the triangulation of methods and data sources. We used multiple data sources by incorporating TBAs, health care professionals, women, community, and religious leaders. Different methods were used in consideration of the environment where participants felt comfortable, making it easy for them to talk.

### Participant recruitment

We used a purposeful sampling technique to identify and collect data from individuals expected to be information-rich for study purposes. With the help of local collaborators, we were able to trace community or religious leaders and TBAs. Community or religious leaders were selected if they knew TBAs and their role in the community but didn’t have a family of TBAs. TBAs were interviewed if they were currently involved in maternal care during childbirth. Then, with the help of community leaders, we were able to get multiparous women who had given birth with the help of TBAs. Community or religious leaders and senior HCPs were purposely selected for key informant interviews to ask about the role of TBA in the community and their integration with the health care system. In this study, health care professionals include nurses/ midwives and doctors working in maternity care in the study area.t

### Data collection tool and procedure

The semi-structured interview and discussion guides were prepared in English by the lead author(AK). After the completion of the development of interview questions and discussion topic guides, the second author(AW) translated it from English to Amharic (national language). The interview guide included questions related to the role of TBAs during pregnancy, childbirth, and after delivery and their integration with health care professionals. Data collectors who had qualitative data collection experience and spoke the local language were recruited. Before the interview, the aim and procedure of the study (including the voice records) and their permission to voice record during interviews and focus group discussions were explained to the study participants. Six in-depth interviews with TBAs and ten key informant interviews (four community or religious leaders and six HCPs) were conducted each lasting 20–45 min.

For pregnant women, we planned FGDs so that participants could freely share their ideas about the role of TBAs for maternal and child health in the community. The discussion topic guide mainly focused on the role of TBAs and the solution they provided when the mothers faced complications in the remote rural community. The local collaborators facilitated the discussion. Two FGDs with nine participants were conducted and recorded after getting informed consent from the study participants. The first discussion and second discussion took 50 min 80 min respectively. Field notes also were taken to record non-verbal observations during the study. The data were collected on March, 2021.

### Data analysis

All voice records and field notes from FGDs and interviews were transcribed verbatim in the Amharic language by data collectors with the help of local collaborators. The accuracy of transcription was checked many times while playing the voice record. Then it was translated into English by research team members. Members of the research team then compared for similarities and differences of translation among themselves. After reaching an agreement on translation, the English version of the transcript was exported to Atlas. ti version 8 software. Thematic analysis was used to analyze the qualitative data based on inductive analysis in line with the study objectives. This thematic analysis was guided by a step-by-step approach to increase trustworthiness [23].

### Trustworthiness

The trustworthiness of the study was achieved by a step-by-step approach. Emerging themes and subthemes were further checked with subsequent interviews and FGDs. The data collectors undertook prolonged engagement with study participants and this ensured that the phenomenon under investigation was fully understood. Again, independent coding and checking of transcripts ensured that the data analysis was credible. Identification codes (ID) were used to present verbatim quotes.

## Result

### Characterstics of the participants

In total, six TBAs were included in the study. They were all females, none of them had recived training but they were recognized as TBAs in the community. The average age of TBAs was 46 years (ranging from 39–65 years). Eighteen pregnant women with an average age of 28 years ( ranging from 25 to 43 years) came to the nearest school in the community and agreed to participate in the study. All pregnant women and TBAs reported that they have no formal education. About their current marital status, 17 of them were married and 7 were single. All of them identified themselves as being Christian.

In total, six HCPs (1 male doctor and 5 female professional midwives and nurses with an average age of 36 years (ranging from 32-41 years) have participated. Additionally, four male community/religious leaders with an average age of 58 years (ranging from 40- 70) were interviewed. All were farmers by occupation and had no formal education.

**Fig. 1:**
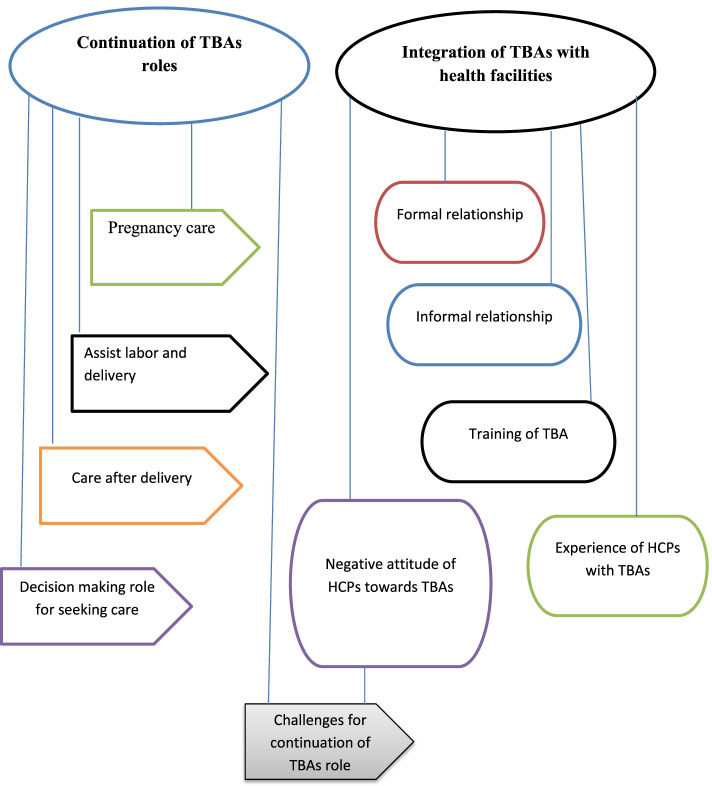
Thematic map on the role of traditional birth attendance regarding the maternal health care and integration with health facilities in West Omo

Two main themes emerged from data analysis about TBAs in maternal health care and their integration with formal health care systems (Fig. **1**).

#### Theme 1: Continuation of TBAs role

##### Sub-theme 1.1: Roles during pregnancy

Description of TBAs role by a religious leader in this study confirmed that their role started by diagnosing pregnancy and advising pregnant women about what could not be done in early pregnancy.

“*TBAs are the first to tell that women get pregnant by looking their physical appearance and give the advice to avoid work load .”* (religious leader)

TBAs stated they provide herbal remdies to manage pregnant women with early pregnancy problems such as nausea and vomiting. TBAs also advised pregnant women late in pregnancy to use herbs that help to reduce labor pain and shorten the duration of labor.

“*Pregnant women call me when they have severe nausea and vomiting and I give them locally produced roots to relive the problem.”* (TBA 1)

*“Near the end of pregnancy, pregnant women will drink boiled tree leaves to relieve pain during labor. When labor is prolonged, the women are advised to chew some of the raw leaves with salt to increase the mothers’ energy to push.” *(TBA 2)

The TBAs are also considered as health care professionals in their community as they know about the presentation of the fetus before labor.

*“ In our locality, they(TBAs) acts as a health professional to identify the presentation of the fetus. They simply observed the abdomen to guess the presentation of the fetus.”* (FGD 2).

##### Sub-theme 1.2: Roles in assisting childbirth

TBAs themselves explained they act as health professionals in assessing the initial stage of labor and assisting childbirth in emergencies, especially in remote rural areas.

“*We take care of the women when women started labor as an emergency at home* or *on the streets in the absence of nearby health facilities or health professionals.” *(TBA3)

However, few TBAs have tried to help women in labor without the required skills. The importance of training TBAs in helping laboring mothers was emphasized by both the community and religious leader.

*“Few TBAs works without experience. Mothers or children may have died. So that they should be trained”* (community leader)

“*In my understanding, TBA is a mother because she is always in the side of a laboring woman. So it is better to support them and to provide training rather than demotivating and punishing them.”* (religious leader)

##### Sub-theme 1.3: Roles after delivery

TBAs not only helped women during childbirth but also after delivery such as preventing bleeding due to retained placenta, and advice on breastfeeding and nutrition for lactating women. However, few of them advised mothers about traditional practices for newborns.

“*TBAs help the women in preventing bleeding by providing herbal remedy at home when the placenta is not dropped*” (FGD 1)

*Eventhough TBAs have provided acceptable advice about breastfeeding and nutritional advice about lactating mothers, few of them advice about milk teeth extraction and uvulectomy which are common malpractices in the community.”* (midwife)

##### Sub-theme 1.4: Decision making for seeking care

The women themselves were not the decision-makers for their healthcare-seeking practice or the place of delivery. All rights of women in the rural communities were at the hand of their husbands. TBAs play a significant role in helping women decide and prepare for pregnancy care or place of delivery.

“*For me, TBAs are mothers of pregnant and non-pregnant women because they are the decision-maker of the women about pregnancy care, complication readiness and preparedness.”* (FGD1)

##### Sub-theme 1.5: Challenges for TBAs role continuation

Previously TBAs played a major role in remote rural communities and were accepted by the community. However, currently, they were discouraged by the local government.

*“Previously in our locality, TBAs work very nice in all activities during pregnancy, childbirth and after delivery. so, please do not discourage them when they help women in labor.”* (FGD 2).

“*Even if TBAs were helping women from the time of pregnancy to postnatal periods, nowadays; some of them had discontinued their role due to demotion by local government.”* (community leader)

TBAs explained that the absence of incentives for their work, lack of training, and shortage of logistics for the care of women during childbirth were the major challenges.

*“We TBAs are working without any training and support from the government. We didn’t get materials needed for a care during childbirth."* (TBA6)

Other TBAs also reported that some health care professionals had negative attitudes towards their role.

*“The health care professionals didn’t have respect for our work while they visit the community during different campaigns."* (TBA5)

#### Main theme 2: The integration of TBA with health facilities

##### Sub-theme 2.1: Formal relationship

HCPs explained that TBAs transferred women to health facilities especially when they encountered difficulty in assisting labor and delivery. The relationship between TBAs and health facilities during care and referral of women varied across the regions in the country.

*“As per my experience, there is no formal relationship with TBAs becaTBAs not only helped women during childbirth but alsouse the government does not interlink the TBAs with the health facility but we integrate with health extension workers.”* (nurse)

*“In some areas of Ethiopia there was a training of TBA and establishment of the formal relationship but it is not well done in all area of the country.”* (doctor).

##### Sub-theme 2.2: Informal relationship

Few TBAs had come to health facilities with the laboring woman as a caregiver (without exposing themselves as TBA) While other TBAs had come along with the woman to the health facility to remove the placenta after childbirth however there is no formal communication with TBAs.

“*As TBA I am afraid to communicate with a health professional. So when I face a problem in attending birth I will go to health center as a family member”* (TBA6).

“*I don’t’ want to go to a health center. But if the placenta is not removed and the mother experience severe bleeding I will go with her.”* (TBA 4)

##### Sub-theme 2.3: Experience towards TBA

The majority of HCPs explained that TBAs play different roles in the community. These include counseling the women to give birth at the health facility, bringing the home-delivered women to the health facility, and to assisting the women in difficult situations such as the absence of transportation and accidental delivery during transportation.

*“I appreciate some of TBAs. Without any training, they helped pregnant women give birth at a health facility or assisted the delivery by themselves when the women were far from reaching the health facility*.” ( midwife)

##### Sub-theme 2.4: Training of TBA

Pregnant women and religious leaders explained that the service of TBAs was uncountable and continuous throughout pregnancy, childbirth, and the postnatal period. They strongly recommended that TBAs should receive training and support from the government.

*“We all are on the side of TBA because they are the nearby mother to pregnant women more than others. Even if we trust them, few TBAs practices are inappropriate and dangerous to the women, so they should be trained.”* (FGD ).

*“I observe that TBAs are always with pregnant women. The family member calls them while the woman is in labor. So it is better to train them in all care of the women.”* ( religious leader).

Health care professionals also recommended the training of TBAs and its impact on reducing feto-maternal death.

*“We should improve the skills of TBA to decrease maternal mortality because the trained TBA could smartly integrate with the health care facilities. In different African countries and even developed countries, they use trained TBAs to decrease maternal mortality.”* (midwife).

## Discussion

This study explored the role of TBAs in maternal care and the problem of integration with health care facilities in remote rural communities of west Omo zone southern Ethiopia. This study indicated that TBAs play an important role in improving maternal health such as managing early pregnancy problems, identifying the presentation of the fetus, diagnosing labor, assisting childbirth, especially in unexpected childbirth, preventing hemorrhage, and promoting healthy messages such as breastfeeding. This finding was supported by the studies reported in Arbaminch [20] and the Afar region [24] in Ethiopia, Ghana [25], Zambia [26], and Pakistan [27].

Additionally, our study highlighted the role of TBAs in decision-making regarding the place of delivery, birth preparedness, and readiness. This practice was highly recommended to be continued. This could be due to the proximity and respectful attitude of TBAs towards the community they serve. However, study reported the husband was the decision-maker for the place of delivery and the decision to seek the service of TBA themselves [26].

In this study absence of incentives, lack of training, and shortage of logistics were reported as the major challenges for the continuity of TBAs' role or integration with the formal health care system. Similar to our finding, a qualitative study in South Sudan revealed that lack of material and supplies for TBAs, insufficient monetary incentives, insufficient/lack of training were reported as challenges faced by TBAs in adopting new roles [17]. On the other hand in another study in Ebonyi state Nigeria provision of incentives and equipment for maternal health care were found to motivate TBAs to refer a client to a health care facility [28].

The integration between TBA and health facilities varied across the region in Ethiopia. In this study, there was no formal relationship between TBA and health care facilities. TBAs have brought women to health facilities only if they faced problems while attending birth like bleeding or retained placenta but they(TBAs) didn’t expose themselves as TBA. TBAs who created a trusted relationship with the community they serve can act as a bridge to strengthen the referral system between the community and the health care system. Eventhough the importance of TBAs' role in referral is universally acknowledged, most health care systems have not developed a formal referral mechanism [14, 17, 20, 26].

## Conclusion

TBAs have continued their role during pregnancy, childbirth, and after delivery despite the existing challenges. They were deciding the place of delivery for pregnant women in this study. There was no integration between TBA and the formal health care system. Lack of training, absence of incentives, and shortage of logistics for fetomaternal care were the major challenges reported. The need for training TBAs has got great emphasis by all study participants and its impact on reducing feto-maternal death was recognized by health care professionals. Therefore, the federal ministry of health should work for the development of TBAs to scale up their skill across all regions in the country.

## Data Availability

The data generated and used for analysis in this study can be accesed from the corresponding author upon reasonable request.

## Abbreviations

TBAs: Traditional Birth Attendants
HCPs: Health care professionals
WHO: World Health Organization
FGDS: Focus group discussions
